# On Some Modeling Issues in Estimating Vaccine Efficacy

**DOI:** 10.1002/pst.2440

**Published:** 2024-09-09

**Authors:** Mauro Gasparini

**Affiliations:** ^1^ Department of Mathematical Sciences “G.L. Lagrange” Politecnico di Torino Torino Italy

**Keywords:** BioNTtech, Covid‐19, incidence rate, Pfizer

## Abstract

I would like to reconsider a recent analysis by Prof. Senn on the statistics of the Pfizer‐BioNTech vaccine trial, to express some different opinions and to clarify some theoretical points, especially regarding the clinical applications of Bayesian statistics.

**Abbreviations**
IRR: incidence rate ratio
VE: vaccine efficacy

## 1 Introduction

This is a reader's reaction to a paper that appeared in this journal [1] authored by Professor Stephen Senn, a very authoritative and widely quoted persona in clinical and pharmaceutical statistics and a personal friend.

I would like to express different opinions on three inter‐related subjects:

1. the general definition of vaccine efficacy (VE from now on) and related modeling issues;
2. the choice of the prior distribution in the Pfizer/BioNTech [2] famous trial;
3. the final Bayesian analysis in the Pfizer/BioNTech [2] trial.

## 2 On the Definition of Vaccine Efficacy

Prof. Senn [1] defines VE as

(1) $$\mathrm{VE}=\frac{\pi_{C}-\pi_{V}}{\pi_{C}}$$

where $\pi_{V}$ $\pi_{C}$ are the probabilities of infection if given the vaccine or the control (often placebo) treatment.

Now, infection is a process developing over time in a population but this definition, although apparently clear and simple, contains no reference to time. Probability of infection in 1 month, say, is smaller than probability of infection over 2 months. A patient randomized right after study begins is much more likely to be infected than a patient randomized toward its end. Definition (1) only works in the idealized situation where each patient is randomized in the same day and observed for the same amount of time, something that is far from how things happen.

The correct definition of VE can be based on representing the data as time‐to‐event data. If infection times are i.i.d. exponential within each group, one can assume the infection processes can be modeled by two homogeneous Poisson point processes: one for vaccinated participants—with intensity $\lambda_{v}$—and an independent one for the participants in the control group—with intensity $\lambda_{c}$. This is the simplest approach and a common one in the epidemiological literature.

The time dimension of each Poisson process is called *surveillance time* and it is measured in person‐years of follow‐up. It is the sum of all durations, participants have been experiencing in the clinical trial. Infected participants contribute with an event at the end of their duration, while noninfected ones contribute just with their follow‐up duration. In the Pfizer‐BioNTech trial considered in the cited references [1, 2], each patient contributes to surveillance time from 7 days after the second dose up until the earliest of the following four endpoints happens: onset of disease, death, loss to follow up or end of study.

A common measure of comparison between two infection processes in Epidemiology is the incidence rate ratio $\mathrm{IRR}=\lambda_{v}/\lambda_{c}$; VE is then defined as

(2) $$\mathrm{VE}=1-\mathrm{IRR}=1-\frac{\lambda_{v}}{\lambda_{c}}$$

which can be interpreted as the average fraction of missed infections (fraction of not infected vaccinated participants who would have been infected if not vaccinated). For example, some standard references [3, 4] are cited.

The definition of VE based on Poisson infection rates is of course a great simplification with respect to more sophisticated mechanistic models such as the SIR (Susceptible/Infected/Removed) model and all its descendants (we have witnessed a plethora of these during the Covid‐19 years) but it has the great advantage of simplifying things to the very core of the matter and of reducing the vaccine measure of effect to the comparison of two incidence rates. Of course, for the simplified model to be literally true, the epidemic evolution has to be in a stable state, an assumption that is very difficult to obtain and verify. This was especially true for the late summer of 2020, when the Pfizer‐BioNTech trial took place; however, the results of the trial were so clear that the simplified model can be considered an effective working tool.

Another fundamental advantage of the infection process approach is that the assumption of Poisson counts fits into the framework, while it would have to be considered an “approximation” if counts were assumed to be binomial (see e.g., Equations (5) and (6) in Prof. Senn's work [1]).

## 3 Into Bayesian Elicitation

My viewpoint differs most from Prof. Senn's in the discussion of the choice of the prior for the parameter θ, the probability of infection in the vaccine group.

I try first to reverse engineering the logic followed by the Pfizer researchers in [2]. To begin with, assume a prior for θ is to be chosen among the beta distributions with first shape parameter ν and second shape parameter ω=1, a constraint to be discussed next:

(3) $$\theta ∼\text{Beta}\left(\nu ,\omega =1\right)$$

In order to elicit the first parameter of the prior, Pfizer researchers choose to anchor θ to reflect a value VE = 30%; in particular, they equated its prior mean $\nu /\left(\nu +1\right)$ to the anchor as follows:

(4) $$\frac{\nu }{\nu +1}=\frac{1-\mathrm{VE}}{2-\mathrm{VE}}$$

and obtain, when VE = 0.3, the result ν=0.7 which can be used for the analysis.

Next, why is it reasonable to impose the constraint ω=1? Simply because this is the smallest value which provides a prior density not exploding to ∞ in the left neighborhood of the value 1. In other words, using the standard definition of beta density recalled in Equation (8) in [1], ω=1 is the smallest value such that

$$\lim_{x\to 1^{-}}\;f\left(\theta \right)<\infty$$

Since we anchored the prior mean to values smaller than 0.5, it would be awkward to assign an almost infinite density to values close to 1; the resulting bi‐modal prior would express a somewhat schizophrenic belief about θ. On the other hand, we do want to keep both ν and ω as small as possible, so that the prior is least informative. To sum up, in the Pfizer article, the resulting choice ν=0.7 and ω=1 is descending from an anchoring argument and from the intention to give a large variance to the prior, but not as large as to produce a bi‐modal prior.

The reader may object the Pfizer paper does not report ν=0.7, but rather ν=0.700102, a “parameter defined to six significant figures” which “does seem somewhat strange” to Prof. Senn [1]. I have a guess for the reason of such a mystery value. Solving Equation (4) with VE = 0.3 in two steps, one gets, rounded to seven significant digits,

(5) $$\frac{\nu }{\nu +1}=0.4117647$$

which of course is solved explicitly as

(6) $$\nu =\frac{0.4117647}{1-0.4117647}=0.7$$

But now, suppose you round 0.4117647 to 0.4118, as done in [1] and possibly in the preparatory work for [2]. Then

(7) $$\nu =\frac{0.4118}{1-0.4118}=0.700102$$

which explains the mystery number! In other words, the somewhat strange number may be the result of a mere rounding error.

**FIGURE 1:**
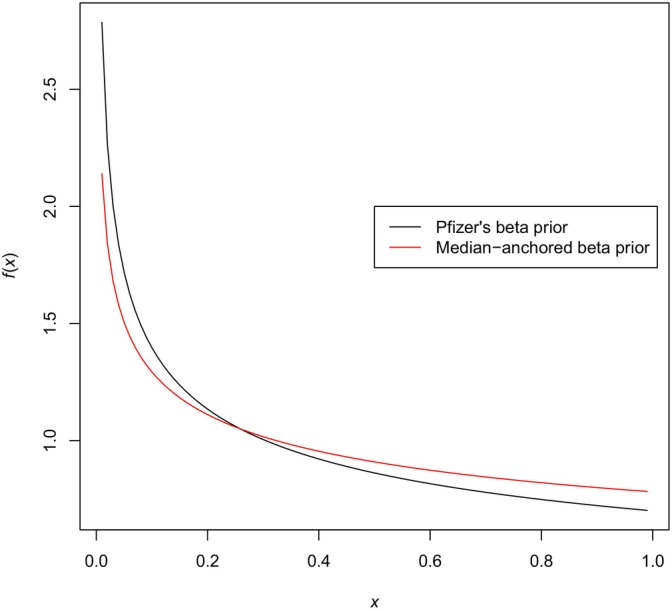
Densities of two beta priors for the probability of infection.

More importantly, let us now consider Prof. Senn's three criticisms to Pfizer's prior specification. His first objection that the mean of a nonlinear function is not the nonlinear function of the mean is of course correct. Pfizer's anchoring argument can be corrected in a very simple way considering that the distribution function of the beta prior (3) is

(8) $$F\left(\theta \right)=\theta^{\nu }\;\left(0\leq x\leq 1\right)$$

so that its median is easily seen to equal $0.5^{1/\nu }$. Anchoring the median of θ—a monotone function of VE—instead of its mean would then provide

(9) $$0.5^{1/\nu }=\frac{1-\mathrm{VE}}{2-\mathrm{VE}}$$

which, solving for VE = 0.3, gives ν=0.7811841. Again, not as round a number as ν=0.7, but the point is that anchoring arguments like these, whatever number they provide, are conceptually simple and defensible. Anchoring using the mean instead of the median would require some numerical work, but conceptually the three approaches are not very different. The difference between Pfizer's prior Beta(0.700102,1) and the prior with anchored median Beta(0.7811841,1) is shown in Figure 1.

The second of Prof. Senn's objections regards the choice of the second parameter of the prior beta distribution. I have already considered the issue in my reverse engineering of Pfizer's elicitation process: the choice ω=1 looks like a reasonable one to avoid bi‐modality and—at the same time—to preserve a large prior variance. The discussion surrounding figure 5 in [1] does not seem so necessary in the light of these simple considerations.

Finally, about the actual value VE = 0.3 used to anchor θ, Prof. Senn writes “The value of 30% is a sort of agreed minimum relevant effect but it does not follow that it should represent prior belief. […] the statement in the protocol that this ‘can be considered pessimistic’ is quite wrong.” I object to that: it is perfectly fine for a Bayesian protocol to be based on a pessimistic scenario in order to convince the skeptical stakeholders. Of course, the true prior belief of the Pfizer researcher was much higher than VE = 0.3, but that value is used as a worst case to protect against criticism of biasing the analysis toward company interests. The use of a skeptical priors in clinical trials has been advocated in a large part of Bayesian literature [5] Some authors [6] express this position arguing for the need of two priors, a *design prior* expressing prior belief and an *analysis prior*—such as the one used by Pfizer—to be used in the actual analysis to reflect neutrality or to comply with regulatory requirements.

## 4 Into Bayesian Posterior Analysis

Modeling VE based on infection rates (Section 2) and prior elicitation on VE (Section 3) finally converge to the main and final point, which is the posterior Bayesian analysis of the results. At the prior stage, it is fine to use the inverse formula

(10) $$\theta =\frac{1-\mathrm{VE}}{2-\mathrm{VE}}$$

for θ, the probability of infection in the vaccine group, as done in Equation (4) above. This is a sensible choice since a priori we have not yet observed the total surveillance times in the vaccine and in the control group and we can assume they are approximately the same. But, at the analysis stage (i.e., a posteriori), the inverse formula used by the Pfizer researchers is

(11) $$\theta =\frac{s_{v}\lambda_{v}}{s_{v}\lambda_{v}+s_{c}\lambda_{c}}=\frac{s_{v}\left(1-\mathrm{VE}\right)}{s_{v}\left(1-\mathrm{VE}\right)+s_{c}}$$

where $s_{v}$ and $s_{c}$ are the total surveillance times of the vaccine and the control group, respectively. This is so because the analysis conducted in [2] is a Bayesian one *conditional* on the total surveillance times and the total number of successes; this is my understanding of what Pfizer researchers mean by “adjusted for the surveillance time.” I personally would question such conditional analysis and include in the Bayesian posterior the uncertainty associated with the total surveillance times and the total number of successes, but this is an issue to be discussed elsewhere. In any case, it should be noted that the final analysis has to take into account the surveillance times in some way, since in the Poisson process sλ is the expected number of events in a time span long s.

In the Pfizer trial, it turned out that $s_{v}=2214$ and $s_{c}=2222$ so that the posterior $\theta ∼\text{beta}\left(8.7,163\right)$ and in particular its 2.5% percentile qbeta(0.025, 8.7, 163) and its 97.5% percentile qbeta(0.975, 8.7, 163) are reversed into the 95% posterior credible interval with lower extreme

$$1-\text{qbeta}\left(0.975,8.7,163\right)\times 2222/\left(\left(1-\text{qbeta}\left(0.975,8.7,163\right)\right)\times 2214\right)=90.3$$

and upper extreme

$$1-\text{qbeta}\left(0.025,8.7,163\right)\times 2222/\left(\left(1-\text{qbeta}\left(0.025,8.7,163\right)\right)\times 2214\right)=97.6$$

rounded to one significant digit. This interval is reported in the original paper [2] and in table 5 of [1] but its derivation is not commented by Prof. Senn, who instead reports a frequentist interval in section 6.

From a practical point of view, it does not make a lot of difference since, as Prof. Senn comments, in the Pfizer trial evidence was overwhelming in favor of the vaccine. For that matter, such strong evidence also makes the discussion on prior elicitation less compelling since, as a referee points out, the selection of prior does not have much impact on the posterior estimate, as long as small values of ν and ω are maintained and several infections are observed.

Nonetheless, it is fair—in the view of similar future trials—to stress the elegance and simplicity of the Bayesian interval, which descends directly from a careful modeling of the infection process and from honest prior elicitation.

## 5 Conclusions

Prof. Senn declares [1] to “often fly under a frequentist flag of convenience,” while the paper was written in honor of a celebrated Bayesian, Andy Grieve, another authority in pharmaceutical statistics. I am aware of the enormous contributions to clinical statistics given by both of them in the last 30 years or so. But I felt the need to correct some of the views in [1] for the reasons which I now try to recap.

The distinction between rates and probabilities is at the core of the statistical profession and it is a very important concept to teach, for example, when trying to educate medical doctors or engineers to the right way to quantify risks. Of course, Prof. Senn is totally aware of this, as also shown by the discussion on evolving infection rates in section 9 of his paper. This makes even more surprising his focus on probabilities of infection, which are parameters varying from patient to patient and not a population measure of treatment effect.

Even though Bayesian statistics has done giant steps toward acceptance in general and in particular in the clinical realm, a lot of skepticism remains in some people who still look very suspiciously at all the supposed magic tricks of prior elicitation and Bayesian updating. Prof. Senn's discussion on prior elicitation is overly complicated and hides the very simple and straightforward reasoning, which may have informed Pfizer researchers. However, it is important to stress there is nothing magic or conspiratorial about striving for prior elicitation in an honest way that most stakeholders, even the most skeptical ones, are expected to approve.

Finally, the derivation and use of Bayesian credible intervals, a good substitute for confidence intervals, should be properly recognized and interpreted.

## Conflicts of Interest

The author declares no conflicts of interest.

## Data Availability

Data sharing not applicable to this article as no datasets were generated or analysed during the current study.
